# Late-stage diagnosis of carcinoid heart disease due to lack of access to health care

**DOI:** 10.1186/s40959-023-00176-z

**Published:** 2023-06-05

**Authors:** Aditi Sharma, Maria E. Fierro, Stella Pak, Keerthi Thallapureddy, Moyosore Awobajo, Dawn Hui, Prince Otchere

**Affiliations:** 1 Department of Medicine, The University of Texas Health Science Center at San Antonio, 7703 Floyd Curl Dr, San Antonio, Texas 78229, 210-567-7000 USA; 2 Department of Neurology, Albany Medical College, 43 New Scotland Ave, Albany, NY 12208, 518-262-5521 USA; 3 Department of Pathology, The University of Texas Health Science Center at San Antonio, 7703 Floyd Curl Dr, San Antonio, Texas 78229, 210-567-7000 USA; 4 Department of Cardio-Thoraic Surgery, The University of Texas Health Science Center at San Antonio, 7703 Floyd Curl Dr, San Antonio, Texas 78229, 210-567-7000 USA; 5 Department of Cardio-Oncology, The University of Texas Health Science Center at San Antonio, 7703 Floyd Curl Dr, San Antonio, Texas 78229, 210-567-7000 USA

## Abstract

Carcinoid syndrome (CS) is a unique constellation of symptoms caused by release of vasoactive substances from neuroendocrine tumors (Pandit et al., StatPearls, 2022). Neuroendocrine tumors are rare with an annual incidence of 2 in 100,000 people (Ram et al., 46:21-27, 2019). Up to 50% of patients with these tumors will develop carcinoid syndrome, which is characterized by symptoms caused by elevated levels of serotonin and most commonly include fatigue, flushing, wheezing, and non-specific gastrointestinal symptoms such as diarrhea and malabsorption (Pandit et al., StatPearls, 2022) (Fox et.al., 90:1224-1228, 2004). Over time, patients with carcinoid syndrome can develop carcinoid heart disease (CHD). CHD refers to the cardiac complications that occur when the vasoactive substances, such as serotonin, tachykinins, and prostaglandins, secreted from the carcinoid tumors. These complications most commonly include valvular abnormalities, but can also present as coronary artery damage, arrhythmias or direct myocardial injury (Ram et al., 46:21-27, 2019). While CHD is not typically an initial feature of carcinoid syndrome, it does eventually occur in up to 70% of patients with carcinoid tumors (Ram et al., 46:21-27, 2019) (Jin et.al., 146:65-73, 2021) (Macfie et.al., 224:665-669, 2022). CHD is associated with significant morbidity and mortality due to the risk of progressive heart failure (Bober et.al., 14:1179546820968101, 2020). In this case, we describe a 35-year-old Hispanic woman in South Texas with undiagnosed carcinoid syndrome for over 10 years that eventually progressed to severe CHD. In this patient’s case, we emphasize how lack of access to healthcare resulted in delay of diagnosis, appropriate treatment, and worsened prognosis in this young patient.

**Supplementary Information:**

The online version contains supplementary material available at 10.1186/s40959-023-00176-z.

## Introduction

Carcinoid syndrome (CS) is a unique constellation of symptoms caused by release of vasoactive substances from neuroendocrine tumors [1]. Neuroendocrine tumors are rare with an annual incidence of 2 in 100,000 people [2]. Up to 50% of patients with these tumors will develop carcinoid syndrome, which is characterized by symptoms caused by elevated levels of serotonin and most commonly include fatigue, flushing, wheezing, and non-specific gastrointestinal symptoms such as diarrhea and malabsorption [1, 3]. Over time, patients with carcinoid syndrome can develop carcinoid heart disease (CHD). CHD refers to the cardiac complications that occur when the vasoactive substances, such as serotonin, tachykinins, and prostaglandins, secreted from the carcinoid tumors reach the right side of the heart, causing destruction of the right-sided cardiac valves. Common complications include coronary artery damage, arrhythmias congestive heart failure, and direct myocardial injury [2]. While CHD is not typically an initial feature of carcinoid syndrome, it does eventually occur in up to 70% of patients with carcinoid tumors [2, 4, 5]. CHD is associated with significant morbidity and mortality due to the risk of progressive heart failure [6]. In this case, we describe a 35-year-old Hispanic woman in South Texas with undiagnosed carcinoid syndrome for over 10 years that eventually progressed to severe CHD. In this patient’s case, we emphasize how lack of access to healthcare resulted in delay of diagnosis, appropriate treatment, and worsened prognosis in this young patient.

### Case presentation

A 35-year-old female with no pertinent past medical history presents to the cardio-oncology clinic for evaluation after a recent diagnosis of Stage IV neuroendocrine carcinoid tumor. The patient admits to having gastrointestinal and cardiac related symptoms for many years. Due to her lack of insurance, she had previously been obtaining care in Mexico. Her symptoms began 12 years ago with abdominal pain, for which she was treated with cholecystectomy in 2010. Despite this intervention, her symptoms persisted and progressed to include nausea and diarrhea. Over time they worsened, and this time were attributed to infectious colitis and treated with antibiotics and ranitidine, again, without resolution of her symptoms. In 2015, she developed cardiac symptoms with fatigue and exercise intolerance. Over the years, she began experiencing more symptoms including headaches, shortness of breath (SOB), intermittent leg swelling, abdominal fullness, and abdominal pain.

**Fig. 1:**
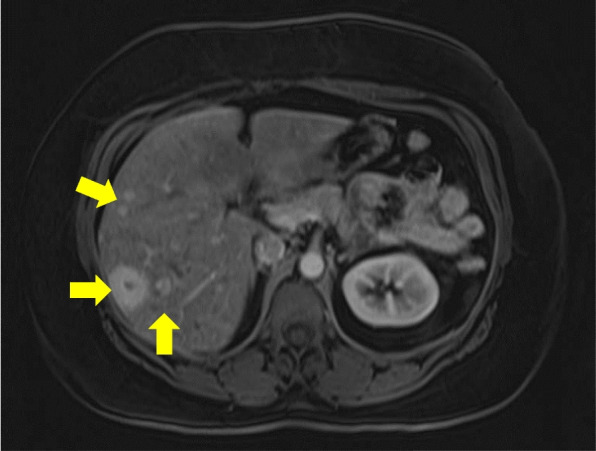
Contrast-Enhanced MRI of abdomen and pelvis. Note the innumerable mild T2 hyperintensities (see the arrows), which correlate to the arterial enhancing, rounded masses throughout the liver

**Fig. 2:**
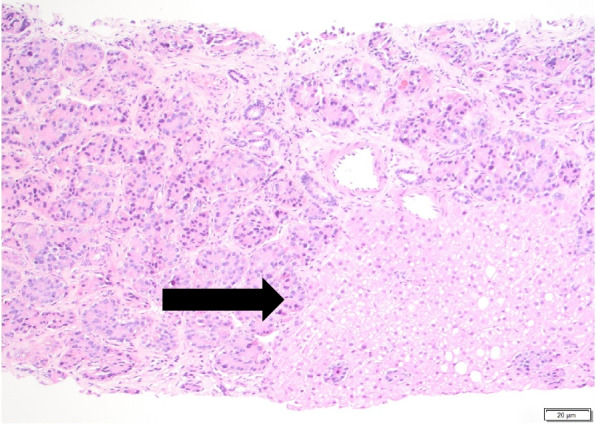
Liver core biopsy showing nested architecture of neoplastic cells composed of small to medium-sized cells with ample eosinophilic cytoplasm (see the arrow) in a background of hepatic parenchyma (hematoxylin and eosin stain, 100 × magnification)

**Fig. 3:**
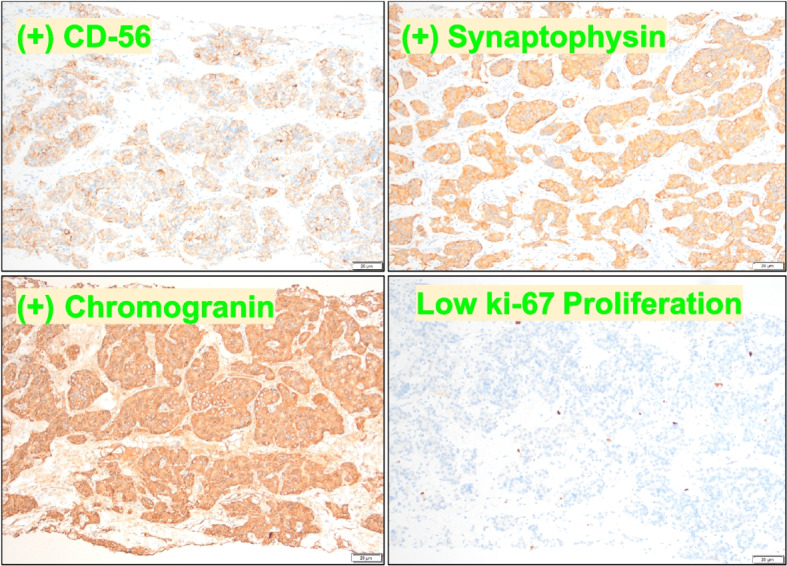
Immunohistochemical stains demonstrated neuroendocrine origin. Positive membranous staining with CD56 (top left, see the text label), membranous and cytoplasmic staining with synaptophysin (top right, see the text label) and chromogranin (bottom left, see the text label) were seen. A low ki67 proliferation index of 2% was present in the tumor cells (bottom right, see the text label) consistent with a diagnosis of well-differentiated neuroendocrine tumor (100 × magnification)

Finally, in September 2021, her abdominal pain became severe enough that she sought care at a local hospital in South Texas. CT abdominal imaging during the visit revealed a mesenteric mass and multiple lesions in her abdomen and liver. MRI was recommended to better characterize the lesions. Unfortunately, due to her uninsured status, the MRI was delayed by another 3 months. In December 2021, she obtained coverage and was able to complete the MRI. Her MRI showed an enhancing lobulated mesenteric mass measuring 2.4 × 2.7 cm and innumerable T2 hyperintense liver lesions which were concerning for liver metastasis (Fig. 1). Two weeks later, she underwent biopsy of the liver lesions, which revealed a well differentiated neuroendocrine tumor (Figs. 2 and 3).

After the diagnosis of neuroendocrine carcinoid tumor was made, the patient established care in the US with oncology and gastroenterology for further workup and treatment. In February 2022, her oncologist started monthly octreotide infusions, which significantly improved her carcinoid symptoms. Seven months after her diagnosis of a carcinoid tumor, her oncologist obtained a transthoracic echocardiogram (TTE) after a new murmur was noted on the patient’s physical exam. The TTE revealed CHD with evidence of right heart failure (enlarged right ventricle and right atria), severe tricuspid valve regurgitation, possible pulmonary valve stenosis, and pulmonary hypertension.

**Fig. 4:**
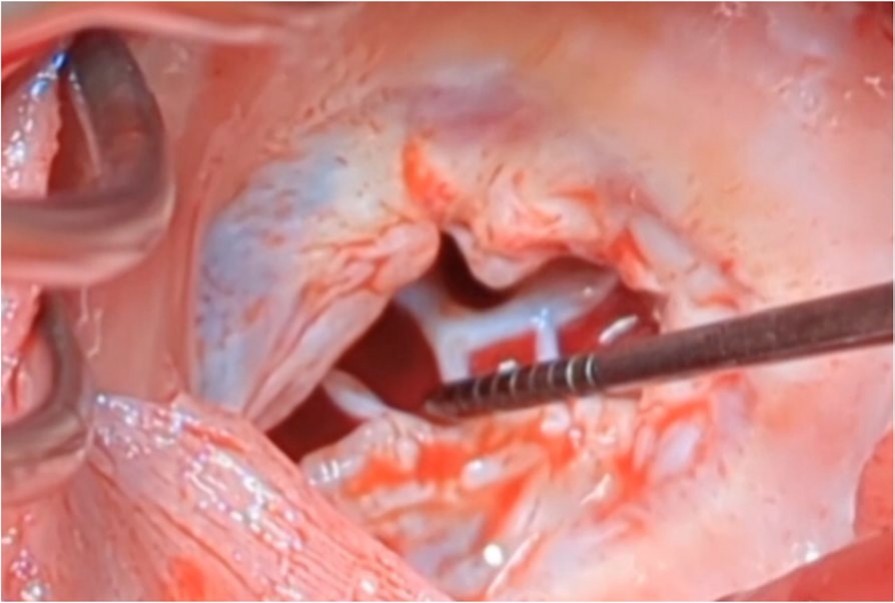
Tricuspid valve replacement with a 31 mm Hancock valve for severe tricuspid valve regurgitation related to CHD and untreated carcinoid syndrome

She was referred to a cardio-oncology clinic in August 2022. On initial evaluation, her physical exam was notable for JVP at 12 cm H2O, giant V waves, 1 + bilateral lower extremity edema, and auscultation of a murmur at the 4th sternal border. A follow up transesophageal echocardiogram (TEE) confirmed the diagnosis with visualization of a thickened tricuspid valve with limited motion and torrential tricuspid regurgitation (Additional file 1 Video 1). She was started on diuretic therapy and referred to cardiothoracic surgery for tricuspid valve replacement. In September 2022, she underwent tricuspid valve replacement with a 31 mm Hancock valve and placement of permanent epicardial atrial and ventricular bipolar leads (Fig. 4). She tolerated the intervention well and had no issues with the carcinoid crisis during her hospital course.

## Discussion

Carcinoid heart disease (CHD) is a major cause of morbidity and mortality in patients with carcinoid syndrome (CS) [6]. As illustrated in this case, development and progression of CHD can lead to the need for major interventions such as valve replacement. Up to 70% of patients with CS will develop CHD [2, 5]. However, as the understanding of this rare syndrome improves, there is growing evidence that highlights ways that CHD can be prevented, one of which is to identify and treat CS in a timely manner. Fluoropyrimidine, such as 5-fluorouracil (5-FU), is associated with an increased risk of cardiac dysfunction, such as dilated cardiomyopathy, arrythmia, congestive heart failure, myocardial injury or inflammation [7]. Avoiding chemotherapeutic agent with known cardiac complications, such as fluoropyrimidine, would be important in the setting of CHD.

Most cases of CS are difficult to diagnose because of the rarity of the condition and the non-specific symptoms it presents with. One study showed that diagnosis of CS can be delayed by up to 7 years [8]. Unfortunately, in our patient’s case, her diagnosis was even further delayed, potentially by up to 12 years. Though diagnosis of CS is challenging, this patient’s delayed diagnosis was likely exacerbated by poor access to the US healthcare system due to her uninsured status. A multicenter retrospective cohort study in Latin America by Uema et al. had findings suggesting that poorer access to care was associated with higher chances of developing CHD [9]. Uema et al. speculates that this difference was secondary to delayed diagnosis and treatment of carcinoid symptoms. Another Latin American retrospective study by Alves et al. further demonstrates the impact of delayed diagnosis, as they found that a longer time from onset of tumor related symptoms to diagnosis of a neuroendocrine tumor was associated with CHD [10]. Lack of insurance results in an additional barrier for patients as most do not have a primary care physician and instead seek care at various hospitals. A Canadian study examining patients with neuroendocrine tumors noted that those with lower socioeconomic status were more likely to require a higher number of diagnostic studies and physician visits for diagnosis [8]. Our patient’s case highlights the importance of improved access to healthcare and timely diagnosis and treatment of CS as ways to prevent development of CHD.

Once the diagnosis of CS is made, prompt treatment with somatostatin analogues, most commonly octreotide, should be initiated. Delayed diagnosis of CS is thought to increase chances of developing CHD because it results in prolonged myocardial exposure to vasoactive substances, specifically serotonin, that induce cardiac valvular fibrosis [4]. While serotonin is likely not the sole culprit for development of CHD, there are clinical studies that show that serotonin and 24-h urinary 5-HIAA were significantly higher in patients who developed CHD in comparison to those who did not [10, 11]. Additionally, development of CHD has been associated with increased morbidity and mortality due to risk of death from progressive heart failure [1, 6]. Dysfunction of the tricuspid and pulmonary valves are often found in this patient population with CHD [12]. It should be noted, however, that while expert consensus opinion recommends octreotide therapy in patients with metastatic carcinoid tumors for prevention of CHD, data is lacking to support this notion. While pre-clinical and clinical data have shown that higher levels of serotonin are associated with CHD, lowering serotonin levels with somatostatin analogues has not been shown to prevent development or progression of CHD [2, 5, 10]. Regardless, therapy with somatostatin analogues should still be initiated for theoretical CHD prevention, reduction of carcinoid symptoms, and for prevention of carcinoid crisis in the perioperative setting [2].

Regular screening for CHD should also be performed in all patients with CS. Guidelines recommend screening with TTE annually for patients *without* cardiac involvement and semiannually for those *with* cardiac involvement [11]. TTE is also the gold standard for diagnosis of CHD [2]. Once CHD is diagnosed, patients should be evaluated every 6–12 months for progression with TTE, NT-pro-BNP, chromogranin A, and urinary 5-HIAA levels to assess for valvular disease, heart failure, and recurrence or worsening of carcinoid malignancy [2, 11].

## Conclusion

As illustrated in our patient’s cause, timely diagnosis and treatment of CS are imperative for prevention of CHD. Once CS is identified, prompt treatment with a somatostatin analogue and regular screening for CHD with TTE should occur. Unfortunately, this patient already had advanced CHD that required tricuspid valve replacement near the time of her initial CS diagnosis. Poor access to the US healthcare system leads to a delayed diagnosis, which resulted in significant morbidity and poor prognosis for this young, 35-year old patient. This case highlights the importance of adequate access to healthcare for timely diagnosis and treatment of CS, appropriate screening for CHD once CS is diagnosed, and appropriate monitoring and therapy when CHD develops.

## Supplementary Information

**Additional file 1: Video 1.** Transesophageal echocardiography demonstrating a torrential tricuspid regurgitation with enlargement of the right atrium.

## Data Availability

Data and materials in the Department Drive can be accessed at any time upon request.
